# Genomic and Phenotypic Characterisation of *Campylobacter jejuni* Isolates From a Waterborne Outbreak

**DOI:** 10.3389/fcimb.2020.594856

**Published:** 2020-10-29

**Authors:** Emma Davies, Marit Ebbesen, Cecilia Johansson, René Kaden, Hilpi Rautelin

**Affiliations:** ^1^Clinical Microbiology, Department of Medical Sciences, Uppsala University, Uppsala, Sweden; ^2^Department of Microbiology, Haukeland University Hospital, Bergen, Norway

**Keywords:** *Campylobacter jejuni*, waterborne, whole-genome sequencing (WGS), outbreak, *in vitro* infection model, gene expression, phylogenetic analyses

## Abstract

*Campylobacter* infections are the leading cause of bacterial gastroenteritis. In Europe, over 246,000 cases are confirmed annually. Infections are often transmitted *via* contaminated food, such as poultry products, but water may be the source of infection as well. The aim of this study was to characterise a selection of *Campylobacter jejuni* human isolates, together with a water isolate, from a waterborne outbreak in Norway in 2019, including human isolates from early, mid-, and late epidemic. The isolates were characterised with whole-genome sequencing, analysing the expression of putative virulence genes and demonstrating the pathogenic potential in an *in vitro* adhesion model using HT-29 cells. All isolates belonged to the multilocus sequence type 1701 and ST45 clonal complex. In the genomic analysis, the water isolate clustered somewhat separately from the human isolates. There was some variation between the human isolates, but the water isolate seemed to display the greatest pathogenic potential, demonstrated by the highest levels of virulence gene expression, adhesion to epithelial cells and IL-8 induction. These results suggest that the water isolate of the study has potential to cause human infections, and that some bacterial changes due to host or environmental adaptation, may occur during a waterborne *Campylobacter* epidemic. This is, to the best of our knowledge, the first study on *C. jejuni* isolates from a waterborne outbreak, including both human isolates and a water isolate, characterised with genomic and phenotypic approaches.

## Introduction

According to the World Health Organization (WHO), foodborne illness poses a global public health challenge, as each year 600 million cases of illness is caused by consuming unsafe food or water (World Health Organization, 2015). *Campylobacter* spp., which cause campylobacteriosis, a diarrhoeal disease in humans, is the most frequently reported foodborne illness in the European Union (EU) creating a financial burden of approximately 2.4 billion euro annually [European Food Safety Authority (EFSA) and European Centre for Disease Prevention and Control (ECDC), 2015]. *C. jejuni* (80%–90%) and *C. coli* (5%–10%) are the two predominant species causing gastrointestinal infections in humans (Blaser, 1997). The most likely source for *Campylobacter* infections is poultry (Skarp et al., 2016), but due to their broad natural reservoir, *Campylobacter* spp. can also be transmitted *via* water. Other known sources include food products, such as unpasteurised milk and contaminated fresh produce (Humphrey et al., 2007). Usually the incubation time ranges between 2 and 5 days (Blaser, 1997). Campylobacteriosis is characterised by symptoms including high fever, headache, nausea, and diarrhoea, which can sometimes be bloody (Blaser, 1997).

*Campylobacter* may cause waterborne outbreaks, when the water source is faecally contaminated either by runoff of surface water after rainfall or *via* a leakage of a sewage pipe in close proximity to the drinking water pipeline (Thomas et al., 1998). In sparsely populated districts, which are abundant in the Nordic countries, groundwater is often used without treatment (Guzman-Herrador et al., 2015). *Campylobacter* infections show seasonal variation in temperate climates, with an increase of the number of reported cases during the warmer summer months (Nylen et al., 2002). This is equally true for waterborne infections, as people are more likely to spend time outdoors, drink untreated water and use water for recreational purposes (Thomas et al., 1998; Schönberg-Norio et al., 2004). Particularly in the Nordic countries, waterborne outbreaks due to contamination of drinking water by *C. jejuni* are common and comprise about one third of all waterborne outbreaks with known aetiology (Guzman-Herrador et al., 2015). During 1998–2012, *Campylobacter* spp. caused 36 waterborne outbreaks in the Nordic countries, infecting over 7,000 people (Guzman-Herrador et al., 2015). In Norway, waterborne outbreaks are reported every year, with the second most common microorganism involved being *Campylobacter* spp. (Hyllestad et al., 2020). The microbiological analysis of water during an outbreak can be challenging, as the contamination is often a short period, and by the time the outbreak is detected, the contamination episode is over (Hänninen et al., 2003; Hyllestad et al., 2020).

Campylobacteriosis appears to be dependent on several virulence factors involving adhesion, invasion and motility. The adhesion to eukaryotic cells is mediated by several proteins, including the *Campylobacter* adhesion to fibronectin protein (CadF), which binds specifically to fibronectin in the cell membrane (Monteville et al., 2003). *Campylobacter cadF* mutant strains have been reported to have a significant decrease, up to 50%, in cellular adherence (Krause-Gruszczynska et al., 2007). *Campylobacter* invasion-associated markers include *iamA*, which has been proposed to be a significant virulence factor, but its function remains unknown. It has been reported that *iamA* is expressed in the majority of invasive, but only in a minority of non-invasive *Campylobacter* isolates (Carvalho et al., 2001). *Campylobacter* may secrete a cytolethal distending toxin (CDT), which is an AB toxin composed of three subunits encoded by *cdtA*, *cdtB*, and *cdtC*. CDT has been proposed to be a notable virulence factor for *Campylobacter*, as CdtB-negative mutants were shown to remain completely inactive in HeLa cell cytotoxicity assays. Similarly, in an animal study using immunocompromised mice, CdtB-negative *C. jejuni* demonstrated impaired invasiveness into blood, spleen and liver tissues (Purdy et al., 2000). It has been suggested that all three CDT proteins are membrane-associated and required for the induction of pro-inflammatory IL-8 (Hickey et al., 2000).

The exact molecular pathogenesis of *Campylobacter* infections remains unclear, as there has been a lack of suitable *in vivo* models that would elucidate the disease in humans (Bereswill et al., 2011). However, *in vitro* infection models showing interactions between bacteria and epithelial cell lines can be used to study the pathogenic potential of *Campylobacter* to cause human infections. The adherence of the bacteria to the cells and the release of pro-inflammatory chemokines, like IL-8 can be quantified *in vitro* (Skarp et al., 2017). To closer examine the genomic characteristics of *Campylobacter* isolates, whole-genome sequencing (WGS) has emerged as an effective method, which offers a high-resolution to observe even minor genomic differences. WGS can increase the understanding of the evolutionary and epidemiological dynamics of *Campylobacter* infections and has the potential to improve surveillance and outbreak detection (Llarena et al., 2017).

In this study, a selection of *C. jejuni* isolates from a recent Norwegian waterborne outbreak was characterised using three approaches: genomic, transcriptional, and *in vitro*. Human *C. jejuni* isolates chosen for the study were selected based on sampling date, including isolates from early, mid-, and late epidemic. In addition, a water isolate, the assumed source isolate of the outbreak, was characterised alike. The aim of the project was to demonstrate possible changes occurring on a genomic, transcriptional and *in vitro* level throughout the epidemic and examine the pathogenic potential of the isolates using WGS, expression of putative virulence genes and an *in vitro* infection assay with human HT-29 colon cancer cells.

## Materials and Methods

### Preparing the Bacterial Isolates

During June 2019, *Campylobacter* caused a large waterborne outbreak in Askøy, north-west of Bergen, Norway, where more than 2,000 inhabitants became infected. The outbreak was due to contamination of the municipal drinking water system (Paruch et al., 2020). For this study, the *Campylobacter* isolates were received from the Department of Microbiology at Haukeland University Hospital, responsible for analysing the faecal samples from the affected inhabitants in the outbreak area. All faecal samples sent to Haukeland University Hospital were originally analysed with real-time PCR (Gastro Panel, LightMix Modular *Campylobacter*, TIB MOLBIOL, Berlin, Germany) and samples positive for *C. jejuni* were further cultivated on selective agar plates. The *Campylobacter* isolates were kept in −80°C prior to transportation to Uppsala on faecal swabs (Fecal Transwab, Medical wire, Corsham, UK). The anonymised isolates were chosen for characterisation based on the stage of the epidemic (**Table 1**). In addition, *C. jejuni* strains NCTC 11168 and 81-176 were included for reference in the experiments. The bacteria were streaked on blood agar plates and incubated at 42°C for 48 h in a microaerobic atmosphere (Campygen, Oxoid, Basingstoke, UK). The isolates were kept in −80°C until needed. All the characterisation experiments were performed on isolates cultured directly from −80°C freezer to keep the passage number low.

**Table 1:** *C. jejuni* isolates included in the study from different stages of the waterborne epidemic.

*C. jejuni* isolate	Source	Sampling date	Accession number	Number of Contigs	N50
2	human, faecal	09/07/2019	JACLAY000000000	91	34,442
9	human, faecal	03/07/2019	JACLAZ000000000	198	15,431
13	human, faecal	25/06/2019	JACLBA000000000	169	21,191
18	human, faecal	20/06/2019	JACLBB000000000	87	50,383
46	human, faecal	12/06/2019	JACLBC000000000	122	26,009
28	tap water	19/06/2019	JACLBD000000000	441	17,675

### Genomics

The *C. jejuni* isolates were cultured for 24 h on blood agar plates prior to DNA extraction. DNA was extracted with the Qiagen EZ1 DNA Tissue Kit (Qiagen Sciences, Germantown, MD, USA), and subjected to WGS. The sequencing itself was carried out by the Swedish Veterinary Institute using MiSeq Desktop Sequencer (Illumina, San Diego, CA, USA). The sequences were assembled into contigs using Geneious (version 8.1.9.) with the Mira plugin (version 1.1.1.) and merging contigs were assembled with Geneious. Read mapping of the virulence genes was done with the raw sequence data to confirm presence of the selected genes that were examined in the *in vitro* and transcriptional experiments. The genetic distance was calculated using the BLASTN algorithm in accurate mode in Gegenees 3.1 with the following parameters: fragment size: 200 bp; step size: 100 bp. Then, a heat map comparing core genomes was constructed using a 20% threshold along with the reference strains NCTC 11168 and 81-176. A neighbour-joining tree based on the core genomes was produced using Geneious (version 8.1.9.). The sequences were deposited to the NCBI database under the project PRJNA656683.

### RNA Preparation and cDNA Synthesis

The bacterial RNA was extracted using the ISOLATE II RNA Mini kit (Bioline Reagents Ltd, London, UK) according to the manufacturer**’**s protocol, with two DNase I treatments (Ambion by life technologies, Carlsbad, USA): on column and on the final RNA preparation. The concentration of RNA was measured using NanoDrop (NanoDrop ND-1000, Thermo Fisher Scientific). The integrity of RNA was tested by running 0.5 µg RNA on a 1% agarose gel with 3% chlorine. For cDNA synthesis, 500 ng of RNA was reverse transcribed using the Maxima First Strand cDNA Synthesis Kit for qPCR (Thermo Fisher Scientific). For the qPCR to determine expression of virulence genes, 1 µl of 10× diluted cDNA was used. Further experiments were performed on four biological extraction replicates.

### Real-Time qPCR

Real-time qPCRs were run in the BioRad CFX96 cycler using the DyNAmo HS SYBR green mix (Thermo Fisher Scientific) according to the manufacturer**’**s protocol. The virulence gene primers were: F-CGCGTTGATGTAGGAGCTAA and R-GCTCCTACATCTGTTCCTCCA for *cdtB*, F-TGGTTTAGCAGGTGGAGGATATG and R-GTTGAAACCCAATTATGGTTTGCATGA for *cadF*, F- TGGAAGTGGAAAATCCGTCCTT and R- GTGCAGCAAACTGAAAAACCACA for *iamA*. *Campylobacter* 16S rRNA primers (Skarp et al., 2017) were included for normalisation.

### *In Vitro* Cell Adhesion Assay and IL-8 ELISA

The pathogenic potential of the selected epidemic *C. jejuni* isolates to cause infections in humans was investigated using an *in vitro* infection model with human HT-29 (ECACC 91072201) colon cancer epithelial cells as prior described (Nilsson et al., 2017). The HT-29 cells were maintained in RPMI 1640 media supplemented with 10% fetal calf serum (FCS) (Gibco by life technologies, Carlsbad, California, US), 2 mM glutamine, 100 U/ml PEST (penicillin, streptomycin) and 100 µg/ml gentamycin (Swedish Veterinary Institute, Uppsala, Sweden). The cells were incubated at 37°C with 5% CO_2_ and were routinely split before reaching approximately 90% confluency, which was determined with microscopy. For the infections, a confluency of 60%–70% was used.

For the adhesion assay, the bacterial suspensions in RPMI 1640 without antibiotics were added to HT-29 cells grown in RPMI 1640 supplemented with 1% FCS to obtain a multiplicity of infection (MOI) of 100. The cells were incubated for 3 h at 37°C. The cells and bacteria were then harvested for downstream analyses. The cells were washed in PBS to remove non-adhered bacteria and lysed (20 mM Tris, pH 7.5, 150 mM NaCl, 0.15% Triton X-100). The lysate was diluted 10× and 100× for qPCR analysis of the 16S rRNA gene together with 10,000× diluted bacteria to determine the adhesion percentage. The qPCR was run with BioRad CFX96 using DyNAmo HS SYBR green mix (Thermo Fisher Scientific) according to the manufacturer’s protocol.

To investigate IL-8 induction, the cell media from the infected cells was removed and diluted four times before analysis using IL-8 ELISA (Thermo Fisher Scientific) according to the manufacturer. The *in vitro* infection assay and IL-8 ELISA were performed on six biological replicates.

## Results

This study characterised human isolates and a water isolate from a waterborne outbreak of *C. jejuni*, which occurred in Askøy, an island northwest of Bergen, Norway during the summer of 2019. According to the local health authorities, approximately 2,000 residents fell ill. This was the largest waterborne outbreak of *C. jejuni* to date in Norway (Hyllestad et al., 2020).

### Phylogenetic Analysis

**Figure 1:**
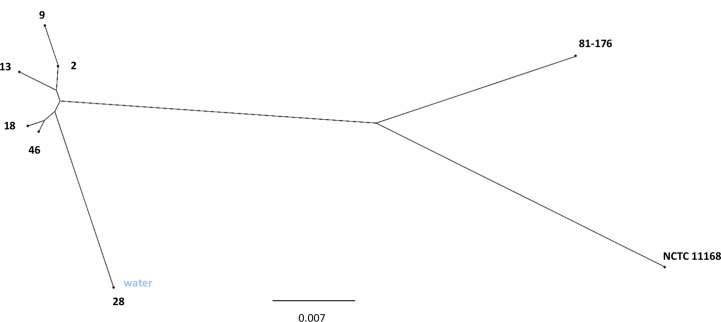
Phylogenetic analysis of *C. jejuni* waterborne outbreak isolates. Neighbour-joining tree based on core genomic similarity displaying the *C. jejuni* human epidemic isolates (2, 9, 13, 18, 46) with the *C. jejuni* water epidemic isolate (28) as well as *C. jejuni* reference strains NCTC 11168 and 81-176.

There are currently more than 10,000 sequence types (STs) that have been typed according to the multilocus sequence typing (MLST) system (https://pubmlst.org). All the isolates of the present study belonged to the same ST45 clonal complex (CC) and were of the same ST1701. Phylogenomic reconstruction based on fragment alignment showed that human epidemic isolates of *C. jejuni* 2, 9, 13, 18, and 46 were closely clustered together (**Figure 1**). The epidemic water isolate clustered close to the human epidemic isolates, but remained still distinguishably separate. The human epidemic isolates had on average between 98.7% and 99.6% similar core genomes, whereas the water isolate differed from the other epidemic isolates with an average of one to two percent (**Table 2**). Accordingly, a slight branching of the water isolate away from the other epidemic *C. jejuni* isolates could be observed in the neighbour-joining tree, also based on the core genomic similarities (**Figure 1**).

**Table 2:** Genomic core similarity of *C. jejuni* isolates.

Isolate	NCTC 11168	81-176	2	9	13	18	46	28
**NCTC 11168**	100	94.8	93	92.4	92.7	93.1	92.9	92.7
**81-176**	94.8	100	94.2	93.5	93.8	94.2	94	93.8
**2 (late)**	93.3	94.4	100	98.7	99.1	99.6	99.4	99.1
**9 (late)**	93.4	94.5	99.8	100	99.4	99.8	99.7	99.4
**13 (mid)**	93.2	94.4	99.7	98.9	100	99.7	99.6	99.2
**18 (mid)**	93.3	94.4	99.6	98.7	99.1	100	99.5	99.1
**46 (early)**	93.4	94.4	99.6	98.8	99.2	99.7	100	99.2
**28 (water)**	92	93	98	97.2	97.5	97.9	97.9	100

Heat-plot illustrating the average genomic core similarities between the waterborne outbreak human isolates (2, 9, 13, 18, 46) and water isolate (28) along with C. jejuni reference strains NCTC 11168 and 81-176. The scores were calculated using a threshold value of 20% for the normalised BLAST-score to exclude genetic material not included in the core genome. Green represents a high similarity and lighter green/yellow represents a lower similarity.

### Expression of Putative Virulence Genes

**Figure 2:**
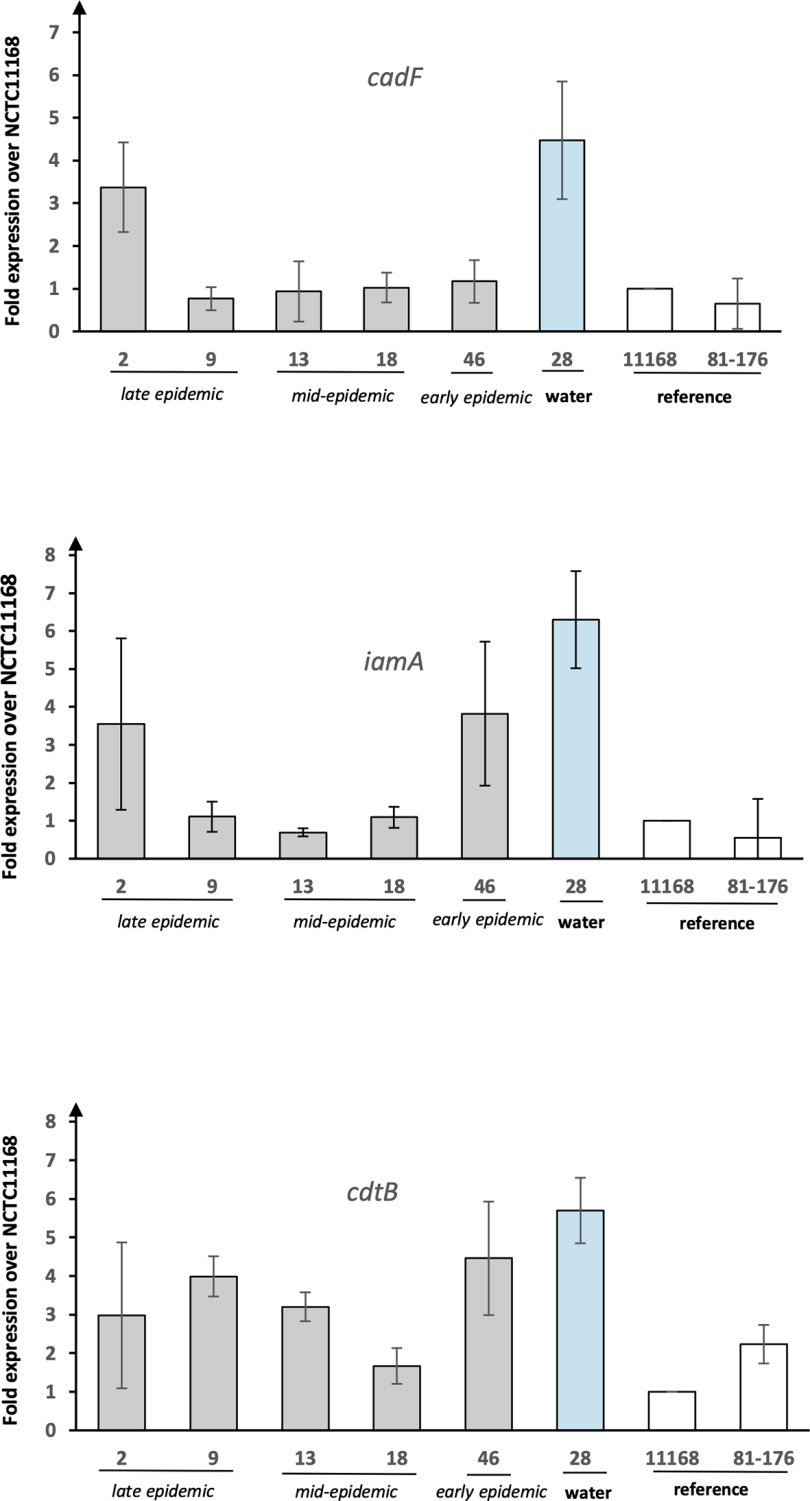
The expression of putative virulence genes from *C. jejuni* waterborne epidemic isolates and two reference strains. Results for the genes *cadF*, *iamA*, and *cdtB* shown as fold increase over reference strain NCTC 11168. Mean values of four biological replicates with error bars indicating standard deviations are shown.

The expression of selected virulence genes (*cadF*, *iamA*, and *cdtB*) was examined with qPCR (**Figure 2**). The presence of all the selected genes was additionally confirmed by read mapping the raw sequence data to virulence gene references. The expression of the genes was demonstrated as fold increase over the reference strain NCTC 11168. The water isolate showed clearly higher expression levels for all the genes analysed as compared to the human epidemic isolates. In comparison to the reference strains, the expression levels of the water isolate were 5–7 fold for all the genes tested. For *cadF*, the human isolates showed in general low expression levels except for the isolate 2 from the late epidemic. The very same human epidemic isolate 2 showed, together with the isolate 46 from early epidemic, higher expression levels than other human epidemic isolates for *iamA*. In general, human epidemic isolates showed higher expression levels for *cdtB* than for *iamA* and *cadF*. Only isolate 18 from the mid-epidemic showed low expression levels for *cdtB*, too.

### *In Vitro* Infection Assay and IL-8 Induction

**Figure 3:**
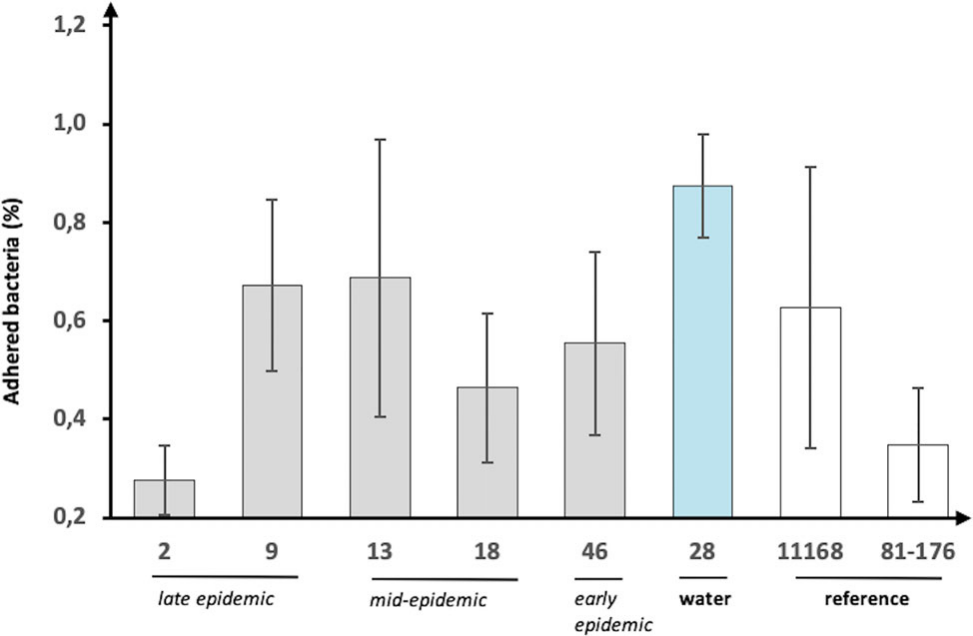
Adherence of *C. jejuni* waterborne epidemic isolates and two reference strains to HT-29 cells. The adherence (%) shown as percentage of the starting inoculum. Mean values of six biological replicates with error bars indicating standard deviations are shown.

The pathogenic potential was examined by infecting HT-29 cells with the epidemic isolates followed by measuring bacterial adherence and the induced IL-8 response. The IL-8 induction results were expressed as fold increase over uninfected (mock) cells, while adherence was calculated as a percentage of the starting inoculum. All the isolates adhered to the HT-29 cells but there was substantial variation between the epidemic isolates (**Figure 3**). Notably, the water isolate showed the highest adherence with almost 1% while the human isolates ranged between approximately 0.3–0.7%. Conversely, human isolate 2, which was from the late epidemic, had the lowest adherence to the cells with 0.28% only. In general, there was no correlation between the sampling date (early, mid-, or late epidemic) and the adhesion level observed.

IL-8 responses also showed variation between the epidemic isolates, but the water isolate had the highest induction of IL-8, with a 7× induction rate over the mock (**Supplementary Figure 1**). There was no correlation between the date of sampling (early, mid-, or late epidemic) and the IL-8 level observed. Isolates 13 and 46 were the only isolates that induced a lower IL-8 response, but had higher adherence compared to the 81-176 reference strain.

## Discussion

Here, a selection of epidemiologically related *C. jejuni* isolates from a 2019 waterborne outbreak in Norway, including human isolates from early, mid-, and late epidemic, and an assumed source isolate from water, were characterised. The results obtained from the genomic analyses supported the epidemiological relatedness, but transcriptional and *in vitro* infection experiments showed interesting differences between the human epidemic isolates and the water isolate. In the phylogenetic analysis, the water isolate clustered somewhat separately from the closely clustered human isolates, and when the expression levels of selected virulence genes were analysed, the water isolate displayed the highest expression levels. In addition, when the pathogenic potential of the epidemic isolates was further analysed in an *in vitro* infection model with HT-29 cells, the water isolate demonstrated evidently higher adherence to the epithelial cells and induction of IL-8.

WGS offers the highest degree of resolution of the typing methods available and is rapidly replacing the traditional molecular methods, such as MLST, in the investigation of *Campylobacter* outbreaks (Llarena et al., 2017). Indeed, the use of WGS-based typing may change the epidemiological understanding of campylobacteriosis, as a study conducted in Denmark suggested, that a large proportion of *Campylobacter* infections are not sporadic, but rather consist of clusters of human infections (Joensen et al., 2020).

In a Finnish study, WGS analysis of *C. jejuni* isolates, which originated from a waterborne outbreak and had been originally characterised using pulsed-field gel electrophoresis (PFGE), revealed some genomic differences among the isolates when re-examined (Revez et al., 2014). According to the results of the aforementioned study, the waterborne outbreak had possibly been caused by at least two closely related *C. jejuni* isolates (Revez et al., 2014). This study, in addition to ours, emphasises the high-resolution discriminatory power offered by WGS, which not only leads to an exact source identification in epidemic investigations, but also allows the detection of minor genomic differences between *Campylobacter* outbreak isolates. Whether the minor changes in outbreak isolates occur during human infections, or at another unknown stage of the outbreak, or if the findings actually refer to the presence of several source isolates, cannot always be answered. Although genomic changes detected by WGS have been shown to occur in *C. jejuni* during human infection (Revez et al., 2013), it cannot be entirely excluded that the genomic variation among the outbreak-related isolates observed in the present study could have been due to an original mixture of several *C. jejuni* isolates. Recently, the potential source of contamination for the outbreak of the current study was described as being of equine faecal origin. This was determined by DNA-based faecal source tracking, which pointed to possible cracks in the drinking water pool (Paruch et al., 2020).

The outbreak isolates of the present study were all of the same ST1701 which belongs to the ST45CC. ST45CC is one of the most common lineages of *C. jejuni*, consisting of generalists frequently detected from various hosts (Dearlove et al., 2016). *C. jejuni i*solates belonging to ST45CC have been reported to be abundant in especially chickens and wild birds (Sheppard et al., 2009). Interestingly, a strikingly analogous *C. jejuni* water isolate originating from a waterborne epidemic in Finland (Revez et al., 2014) was detected. This particular water isolate belonged to ST45, the founder ST within ST45CC, and shared almost 98% core genome similarity to the *C. jejuni* water isolate of the present study (data not shown) although isolated almost 20 years prior the Norwegian isolate described here. It remains to be studied if certain lineages within ST45CC are more successful than others in causing waterborne outbreaks.

All of the *C. jejuni* epidemic isolates tested were able to adhere to and induce an IL-8 response in the HT-29 cell line and thus showed pathogenic potential to cause infection in humans. However, there was variation between the epidemic isolates in their pathogenic potential and interestingly, the water isolate demonstrated the highest adhesion level to the epithelial cells as well as the highest induction rate of IL-8. We have recently studied *C. jejuni* and *C. coli* water isolates in order to better understand characteristics needed for water survival and the potential to cause waterborne infections. We have identified both genomic and phenotypic differences between *C. jejuni* and *C. coli* water isolates (Nilsson et al., 2017; Nilsson et al., 2018b) and suggested that certain *Campylobacter* isolates could have the potential to survive better in water (Nilsson et al., 2018a). However, the water isolates studied prior were not known to be associated with any human infections. In fact, the *C. jejuni* water isolates in the previous study had considerably lower levels of IL-8 induction and cellular adherence to HT-29 cells compared to the NCTC 11168 reference strain (Nilsson et al., 2017), which was also used in the present study. Interestingly, in the current study, the assumed source isolate of the waterborne outbreak was highlighted both in the *in vitro* infection assays and virulence gene expression analyses showing the highest pathogenic potential *in vitro* as compared to the human epidemic isolates and the reference strains. Thus, in addition to the earlier investigations showing the epidemiological relatedness of the water isolate in time, location and source, the present findings further support its role in the outbreak.

In the present study, we had the opportunity to study the genomic, transcriptional and phenotypic variation within epidemiologically related *C. jejuni* isolates from a recent waterborne outbreak. The study offers insight into the similarities, but also the differences between the outbreak isolates and highlights the pathogenic potential of the water isolate. To our current knowledge, this combination of genomic and phenotypic approaches in analyses of human and water outbreak isolates is unique.

## Data Availability Statement

The sequence data used in this study can be found online at: https://www.ncbi.nlm.nih.gov/bioproject/PRJNA656683.

## Author Contributions

Conceptualisation: HR. Methodology: CJ and RK. Validation: CJ and HR. Formal analysis: ED and RK. Investigation: ED. Resources: ME and HR. Writing – original draft preparation: ED. Writing –review and editing: ED, ME, RK, and HR. Supervision: RK and HR. Project administration: HR. Funding Acquisition: HR. All authors contributed to the article and approved the submitted version.

## Funding

This work was supported by ALF grants from the Uppsala University Hospital, Uppsala, Sweden. The funder had no role in study design, data collection and interpretation, or in the decision to submit the work for publication.

## Conflict of Interest

The authors declare that the research was conducted in the absence of any commercial or financial relationships that could be construed as a potential conflict of interest.
