# Supplementary material for: Genomic and Phenotypic Characterisation of Campylobacter jejuni Isolates From a Waterborne Outbreak
Source: Front Cell Infect Microbiol. 2020 Oct 29;10:594856. doi: 10.3389/fcimb.2020.594856 (PMC7658296; doi:10.3389/fcimb.2020.594856)
Supplement: Supplementary file 1 [file DataSheet_1.pdf]

Supplementary Material

1.1 Supplementary Figures

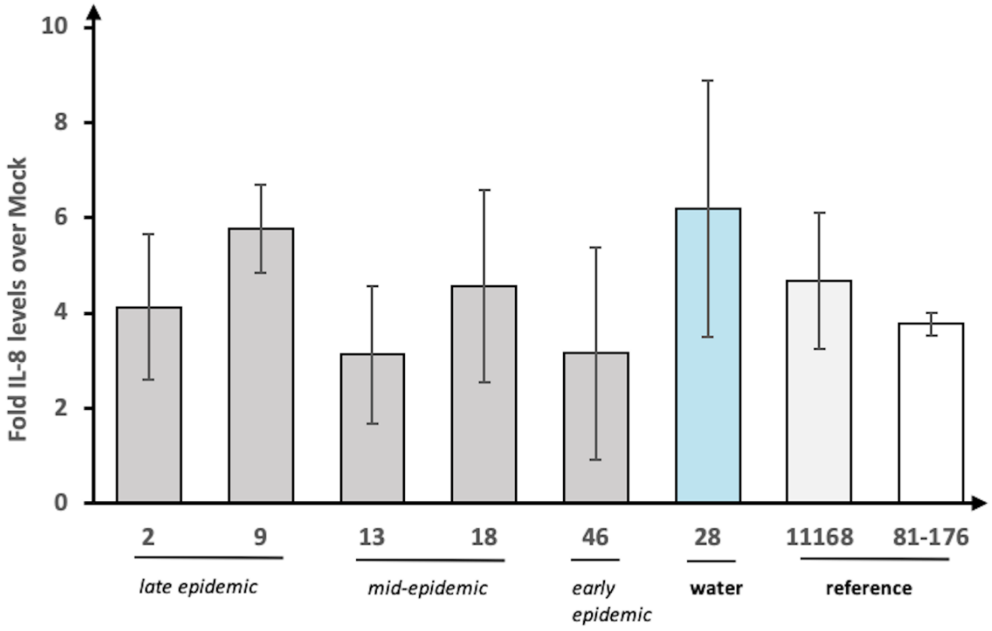

Supplementary Figure 1. IL-8 induction of *C. jejuni* waterborne epidemic isolates and two reference strains. IL-8 production shown as fold increase over uninfected cells. Mean values of six biological replicates with error bars indicating standard deviations are shown.
